# Cluster Headache and Associated Risk Factors: A Systemic Review and Meta-Analysis

**DOI:** 10.7759/cureus.19294

**Published:** 2021-11-05

**Authors:** Abdulateef Sayed A Elbadawi, Abdulmajeed Faisal A Albalawi, Ali K Alghannami, Fahad S Alsuhaymi, Atheer M Alruwaili, Faisal A Almaleki, Maram F Almutairi, Khuloud H Almubaddil, Maryam I Qashqari

**Affiliations:** 1 Preventive Medicine and Public Health, Ministry of Health, Tabuk, SAU; 2 Radiology Department, Maternity and Children's Hospital, Tabuk, SAU; 3 Emergency Department, King Fahad Specialist Hospital, Tabuk, SAU; 4 Al-Dawaa Medical Services, Al-Dawaa Medical Services Co. Ltd, Hafar Al Batin, SAU; 5 Medicine Department, Taibah University College of Medicine, Taibah, SAU; 6 Family Medicine Department, Ministry of National Guard Health Affairs, Riyadh, SAU; 7 Medicine Department, College of Medicine, Imam Mohammad Ibn Saud Islamic University, Riyadh, SAU; 8 General Practice, King Abdullah Medical Complex, Jeddah, SAU

**Keywords:** head trauma, sex discrimination, alcohol, drinking, tobacco, smoking, family history, cluster headache

## Abstract

Cluster headache (CH) has always been associated with several risk factors, including hereditary, environmental, and lifestyle habits. This study focuses on important risk factors, including family history, smoking, alcohol consumption, male predominance, and head trauma associated with CH. The present study aimed at investigating the available literature on cluster headaches and evaluating their associated risk factors. A systematic data search was designed, and scientific data were collected from renowned databases, including PubMed, Cochrane, Embase, Cumulative Index of Nursing and Allied Health Literature (CINAHL), and Google Scholar. Thirty-two studies were selected to execute a systemic review, and 26 studies, consisting of 6,065 CH patients, qualified for a meta-analysis. Statistical analyses were conducted by using MedCalc, version 16.8.4; (MedCalc Software, Ostend, Belgium; http://www.medcalc.org) and Rapidminer statistical software, version 9.6.0 (www.rapidminer.com). We conclude the evidence of family history, smoking, alcohol consumption, male predominance, and head trauma are associated with cluster headaches. However, sex discrimination in familial cases needs to be revisited because of the female predominance reported by familial history and CH association studies.

## Introduction and background

Cluster headache (CH) is an extreme neurological pathology of primary headache, also called a "suicide headache" [1-2]. Its distinguishing feature is a severe unilateral headache that lasts from 15 minutes to two hours, with up to eight daily episodes [3]. CH is substantially a rare pathology, with a one in 1,000 prevalence [1]. However, its prevalence varies with a geographical variation. It has remission episodes that occur weekly to monthly [3]. The particular trigeminal autonomic characteristics, the predominance of the male gender, and circadian rhythm are the clinical characteristics [1, 4]. CH leads to poor quality of life because of its severe pain intensity and frequent episodes, affecting the patients' well-being and socio-economic condition [5]. The last decade was prominently progressive that generated thoughtful scientific literature to explore the pathophysiology of CH and its management strategies [1-3]. The extensive scientific literature on CH-associated risk factors includes family history (FH), smoking, alcohol consumption, gender discrimination, and head trauma.

Genetic characteristics and familial linkage are the core components of CH patients. Extensive literature has been published to support the gene linkage in CH patients, such as physical characteristics and leukocyte antigens connection [3]. However, family history also depends on environmental factors [1]. Alcohol consumption and smoking are considered as triggering factors and increase the possibility and severity of CH [2, 6]. Gender discrimination is another associated risk factor because of the connection of sex hormones to CH. This also discriminates in disease presentation, severity, and pain intensity [7]. Post-traumatic headache (PTH) cluster headache has been linked with a chronic variant of CH disorder [8].

We designed this comprehensive systemic review and meta-analysis to investigate the available literature on cluster headache and evaluate its associated risk factors.

## Review

Methods

Two authors were designated to execute an extensive search of scientific studies independently. Well-established digital databases were used, including PubMed, Cochrane, Google Scholar, Embase, and Cumulative Index of Nursing and Allied Health Literature (CINAHL). Data from 1990 to July 2021 were extracted by defined keywords without age, population, and ethnicity filters. The defined unique keywords were listed to avoid any data loss (cluster headache and family history, OR cluster headache and family, OR cluster headache and familial, OR cluster headache and smoking, OR cluster headache and tobacco, OR cluster headache and drinking, OR cluster headache and alcohol, OR cluster headache and male, OR cluster headache and gender, OR cluster headache and male sex, OR cluster headache and trauma, OR cluster headache and head trauma). The reference section of selected articles was also analyzed in case any data were overlooked.

The established inclusion criteria were the scientific studies that reported the interaction between (1) family history and CH (studies reporting probands), (2) alcohol consumption/alcohol drinking and CH, (3) smoking and CH, (4) gender discrimination/male predominance and CH, and (5) head trauma and CH.

The exclusion criteria were (1) non-conclusive studies preliminary studies, (2) review and opinion articles, letters to the editor, and case reports, (3) poster or scientific presentations, and (4) non-English articles.

The primary outcome measures included the interaction of defined risk factors with CH: association of family history with CH; the impact of smoking on CH; the impact of alcohol drinking/alcohol consumption on CH; the impact of gender discrimination on CH, and the impact of head trauma on CH. The secondary outcome was not defined in advance.

**Figure 1:**
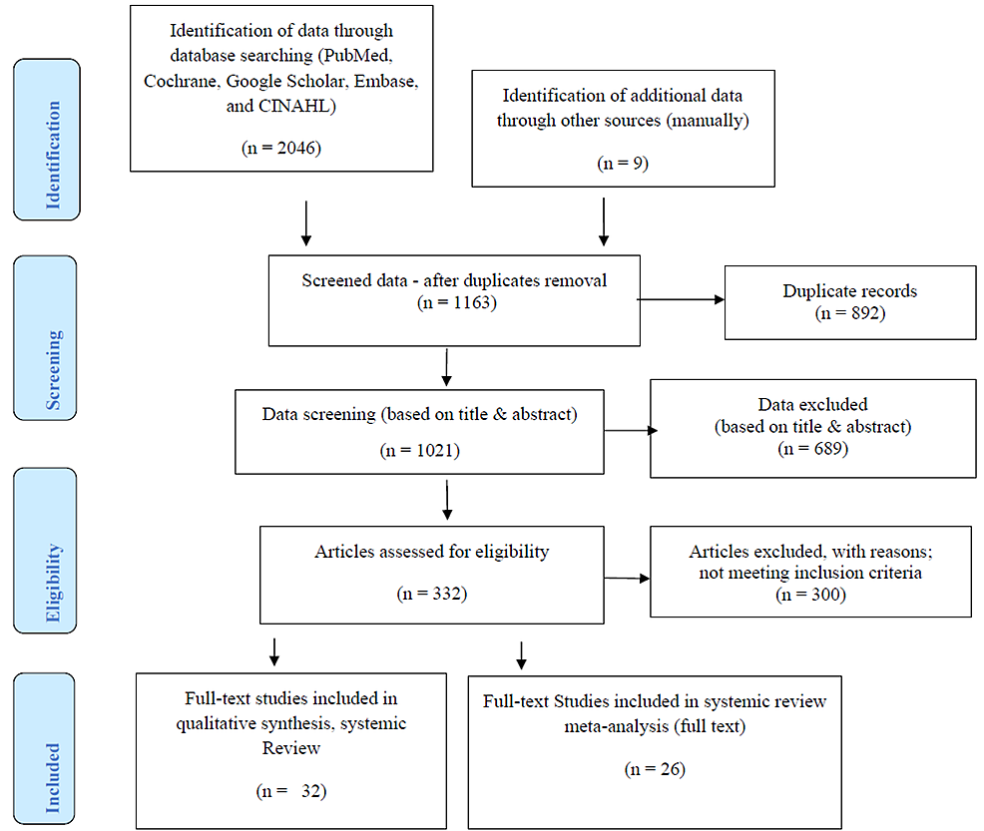
Summary of study selection process CINAHL: Cumulative Index of Nursing and Allied Health Literature

The designated researchers independently screened the data if they fulfilled the established inclusion criteria. Any differences in the study selection process were discussed and finalized by consensus. Data extraction was performed twice independently as per the defined inclusion criteria and Medical Subject Heading (MeSH®) terms to avoid any possibility of data loss and risk of bias [9]. A comprehensive overview of all identified studies formulated is in Tables 1-2. Table 1 represents the studies lined up for systemic review and meta-analysis, while Table 2 represents systemic review studies. All selected studies were based on our defined scope. The Preferred Reporting Items for Systematic Reviews and Metanalyses (PRISMA) statement was followed for data extraction until data selection (Figure 1) [10].

**Table 1:** Overview of Selected Studies Included in the Systemic Review and Meta-Analysis CH: cluster headache; F: female; FH: family history; HCRTR2: hypocretin receptor-2; M: male; OR: odds ratio; PTH: post-traumatic headache

S. no	First author, year, & reference no.	No. of patients	Conclusion	FH prevalence (N, %)	Smoking (%)	Alcohol drinking (%)	M/F ratio OR (N, %)	Head trauma	Proband reported (%)
1	Russell 1995 [11]	421	Suggested for genetic link	_	_	_		_	6.8
2	El Amrani et al. 2002 [12]	186	Accurate inheritance mode was not identified	_	_	_		_	10.8
3	Taga et al. 2015 [13]	785 (F)	Genetic factors connected to early disease onset in females; familial cases have low male-to-female ratio	_	_	_	_	_	5.1
4	Cruz et al. 2013 [14]	36	Supported the evidence of familial aggregation in CH	_	_	_	_	_	20.8
5	Maytal et al. 1992 [15]	35	CH episodes increased with time	_	_	_	_	_	8.6
6	Dong et al. 2013 [16]	13	Low prevalence in Chinese population	_	_	_	_	_	6.7
7	Haane et al. 2013 [17]	10	CH pathophysiology not correctly identified by nociception specific blink reflex	_	_	_	_	_	12.5
8	Leone et al. 2001 [18]	220	Supports the genetic connection	_	_	_	_	_	20.0
9	Lin et al. 2004 [19]	104	Low CH prevalence	_	_	_	79	_	5.8
10	Rainero et al. 2008 [20]	109	Functional activity was influenced by HCRTR2 and V308I genes	_	_	_	_	_	4.6
11	Sjöstrand et al. 2005 [21]	55	Supported a genetic connection	_	_	_	_	_	2.0
12	Steinberg et al. 2018 [22]	500	Variation of disease severity based on daily living	_	80.1	_		_	11.2
13	Bhargava et al. 2014 [23]	30	Low frequency of family history	0	53.3	10	_	_	_
14	Chung et al. 2021 [24]	250	Clinical features do not significantly differ between smokers and non-smokers. Smoking history significantly associated with disease onset age, gender, and seasonal rhythmicity	_	60.8	59.3	83.6	_	_
15	Al-Hashel et al. 2019 [25]	46	High M/F ratio, less evidence of family history; smoking as a strong risk factor of disease severity	3 (6.5)	63	6.5	94.5	_	_
16	Ko et al. 2021 [26]	82	Less prevalence of chronic cases	1 (1.3)	55.7	14.1	83.7	_	_
17	Imai et al. 2019 [27]	131	Alcohol drinking might interfere with the management response	_	53	75	76	_	_
18	Moon et al. 2017 [2]	200	High M/F ratio, low prevalence of chronic cases	_	75	_	88	_	_
19	Ferrari et al. 2013 [28]	200	Smoking associated with severe CH presentation	_	81	_	86	_	_
20	Lund et al. 2019 [29]	400	Unhealthy lifestyle associated with CH	_	48.3	_	_	_	_
21	Grangeon et al. 2020 [8]	553	PTH-­CH probably suffer from chronic variant	_	_	_	73.0	26/553 (4.8%)	_
22	Hannerz 1997 [30]	27 (F)	Smoking was significantly associated with CH	_	89	_	_	_	_
23	Manzoni 1999 [31]	374 (M)	Suggested for improved living habits	_	89.2	_	_	_	_
24	Levi et al. 1992 [32]	51 (M)	Alcohol drinking and smoking associated with CH in male patients	_	97	_	_	_	_
25	Rozen et al. 2018 [33]	1134	Tobacco consumption linked with CH	_	88	_	_	_	_
26	Donnet et al. 2007 [34]	113	Male sex and smoking linked with chronic CH	_	88	_	_	_	6 (5.5)

**Table 2:** Overview of Selected Studies Included in the Systemic Review CH: cluster headache

S. no	First author, year, & reference no.	No. of patients	Conclusion
28	Torelli et al. 2003 [35]	30	Male:female ratio: 1.4:1 (familial cases). Genetic factors linked with female gender.
29	Lambru et al. 2010 [36]	200	Traumatic head injuries were more frequently seen in CH patients with heavy alcohol, tobacco, and coffee consumption.
30	Sjöstrand et al. 2010 [37]	114	Finding supported the evidence of gene interaction and lifestyle habits, including smoking and alcohol drinking. Head trauma cases were also reported in CH patients.
31	Schürks et al. 2006 [38]	246	Alcohol consumption was not predictably different in CH patients from the non-CH population.
32	Manzoni 1998 [39]	482	Alteration of gene ratio in the past decade from 6.2:1 to 5.6:1, 4.3:1, 3.0:1, and 2.1:1 before 1960’s, 1960s, 1970s, 1980s, and 1990s, respectively. Smoking habit might be the reason.

Results 

Characteristics of Study Selection

A total of 2,055 potentially eligible records were extracted in the initial data retrieval process. During the screening process, 892 records were eliminated due to duplication, and 689 were eliminated based on the study title and abstract. The remaining 332 studies were reviewed, 300 articles were excluded for not meeting inclusion criteria. Finally, 32 studies were selected in qualitative synthesis, systemic review, and 26 studies for systemic review and meta-analysis. The PRISMA process describes the search process for, and identification of studies is shown in Figure 1. Our selected studies clearly demonstrate the impact of listed risk factors, including family history, smoking, alcohol drinking, gender discrimination (M/F ratio), and head trauma, as described in Table 1. Table 2 represents the overview of descriptive studies that reported the CH-associated risk factors and their impact.

Statistical Analysis

**Figure 2:**
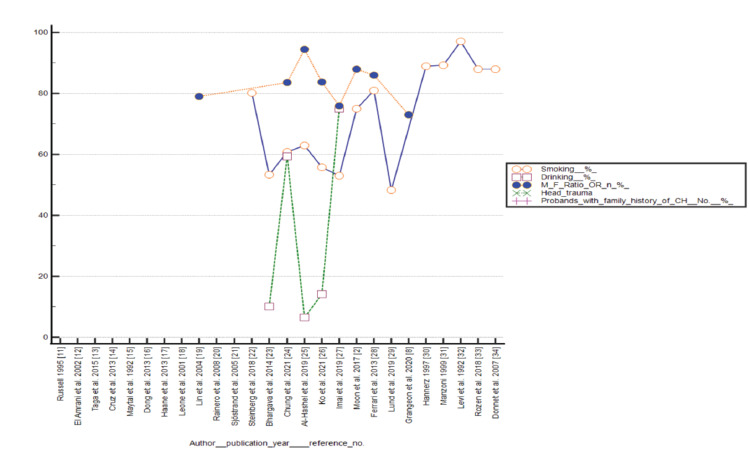
Distribution of data included in the systemic review and meta-analysis studies [2, 8, 11-34]

MedCalc (version 16.8.4; MedCalc Software, Ostend, Belgium; http://www.medcalc.org) and Rapidminer statistical software, version 9.6.0 (www.rapidminer.com) were used to perform all statistical analyses. The MedCalc software was used to calculate the confidence level against each study, test for heterogeneity, and forest plot data presentation against primary outcomes, including smoking, alcohol drinking, and male-to-female ratio. Funnel plots were examined to analyze any publication bias against smoking, alcohol drinking, and male-to-female ratio. The Rapidminer statistical software was used for data presentation of probands with family history. Studies that reported head trauma injuries in relation to CH were less in number for statistical analysis. The distribution of data included in the systemic review and meta-analysis studies is represented in Figure 2.

Primary outcomes

Impact of Smoking on CH

**Figure 3:**
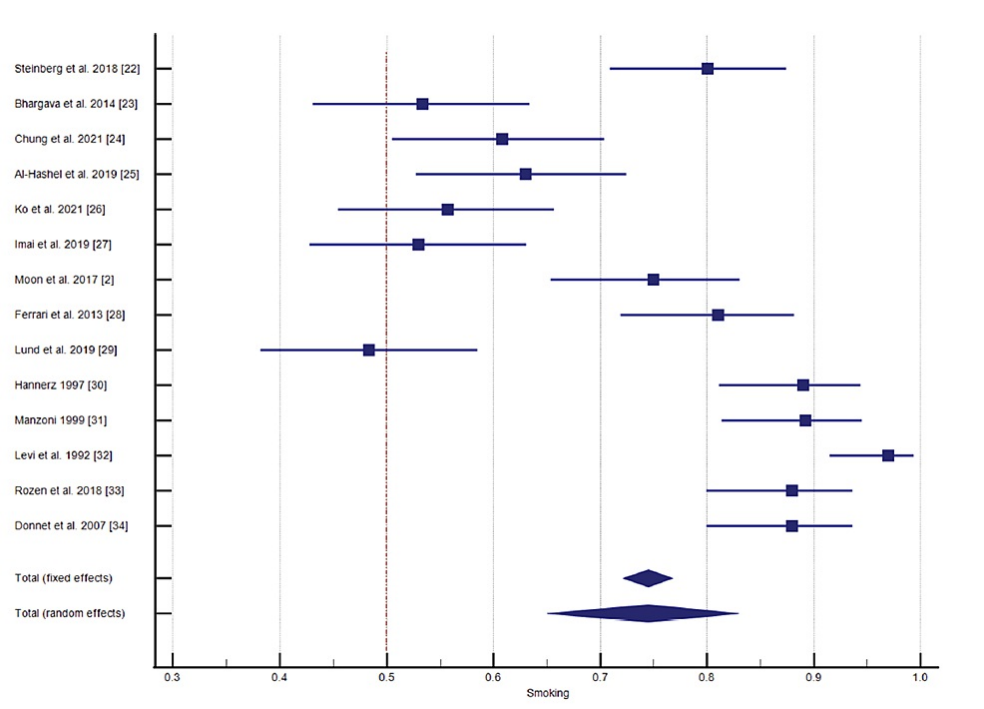
Impact of smoking on cluster headache (CH) [2, 22-34]

**Figure 4:**
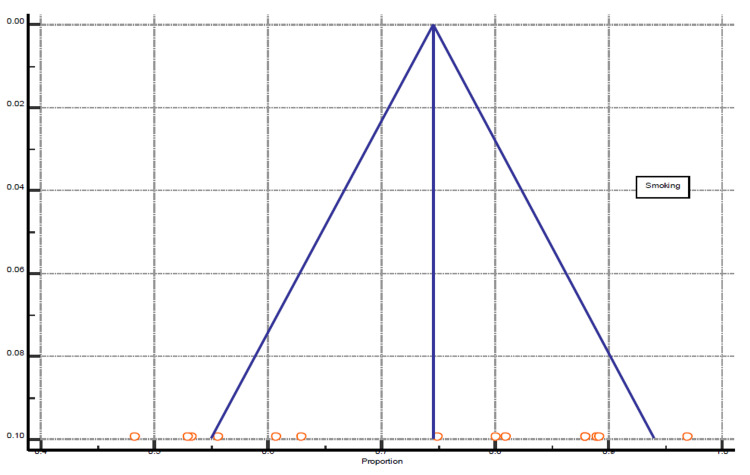
Funnel plot presentation of smoking

Among the 26 selected studies, 14 studies reported the connection of smoking with CH. The significance level, P < 0.0001, 93.66% I^2^ inconsistency, and 95% confidence interval were calculated and is represented in Table 3 and Figure 3 for the test of heterogeneity of smoking. The funnel plot presentation of smoking shown in Figure 4.

**Table 3:** Test for Heterogeneity of Smoking CI: confidence interval; DF: degrees of freedom; I^2^: I-squared value

Q-value	DF	Significance level	I^2^ (inconsistency)	95% CI for I^2^	Total (fixed effects)	Total (random effects)
205.0901	13	P < 0.0001	93.66%	90.96 to 95.55	72.147 to 76.757	65.021 to 82.939

Impact of Alcohol Drinking/Alcohol Consumption on CH

**Figure 5:**
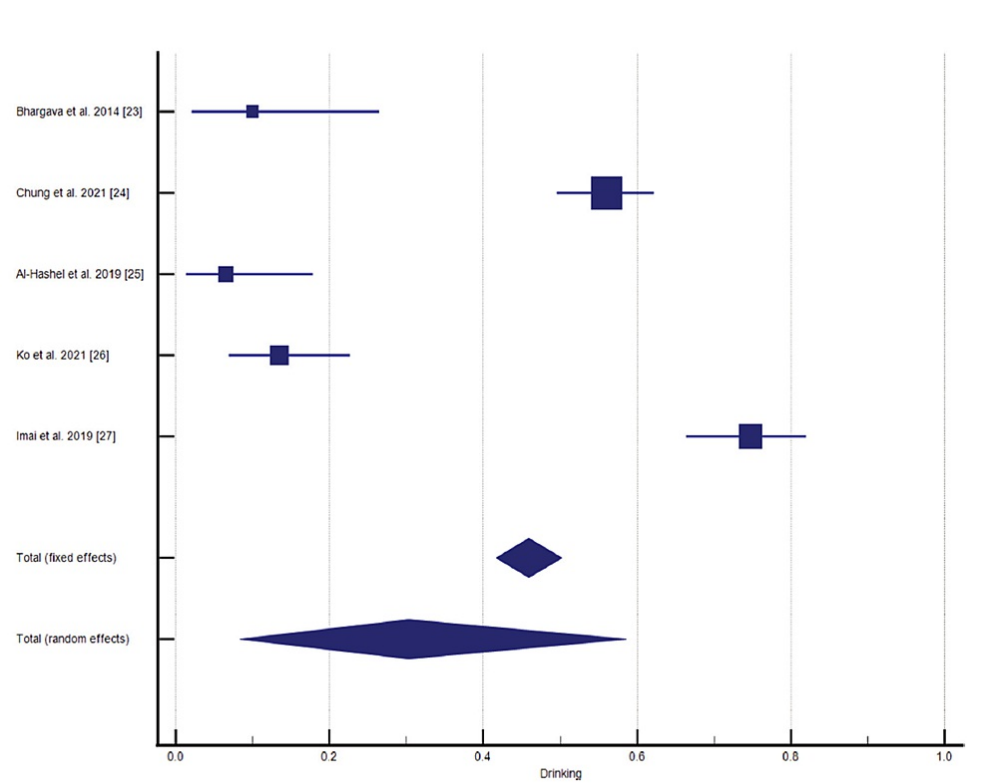
Impact of alcohol drinking/alcohol consumption on cluster headache (CH) [23-27]

**Figure 6:**
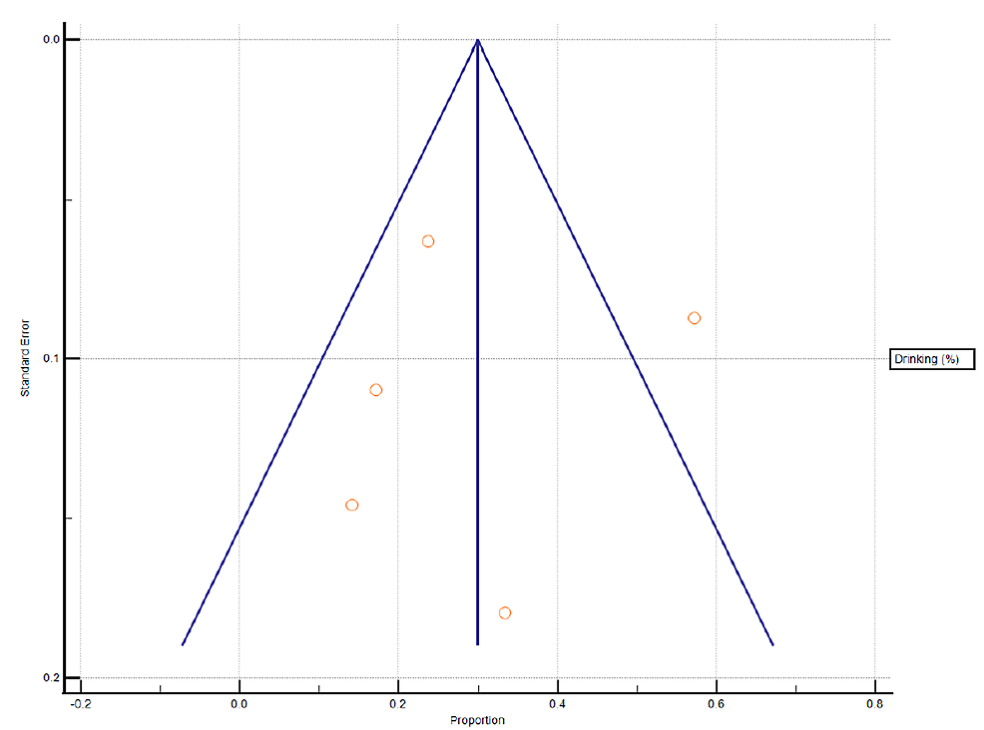
Funnel plot presentation of alcohol drinking

Five studies from the selected data reported alcohol consumption in CH patients and its impact on disease prevalence and severity. A statistical analysis reported P < 0.0001 significance level, 97.54% I^2^ (inconsistency), and 95% confidence interval (Figures 5-6, Table 4).

**Table 4:** Test for Heterogeneity for Alcohol Drinking CI: confidence interval; DF: degrees of freedom; I^2^: I-squared value

Q-value	DF	Significance level	I^2^ (inconsistency)	95% CI for I^2^	Total (fixed effects)	Total (random effects)
162.4015	4	P < 0.0001	97.54%	96.05 to 98.46	42.672 to 50.212	8.391 to 58.592

Prevalence of Male-to-Female Ratio in CH Patients

**Figure 7:**
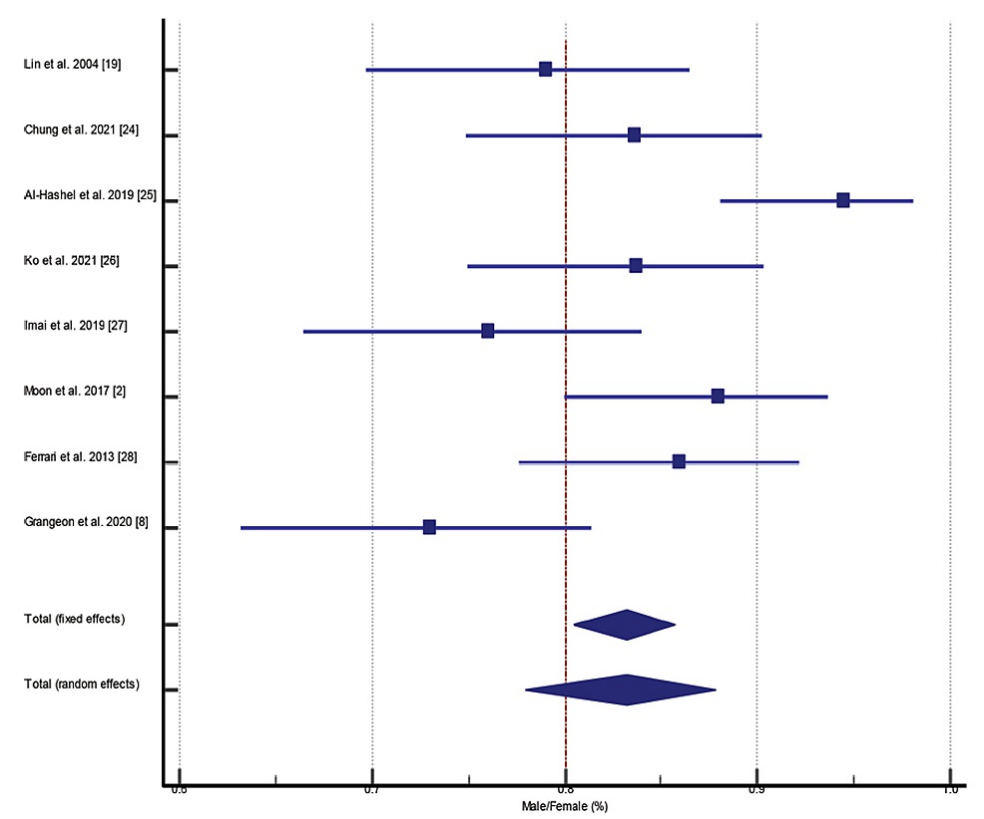
Prevalence of male-to-female ratio in cluster headache (CH) patients [2, 8, 19, 24-28]

**Figure 8:**
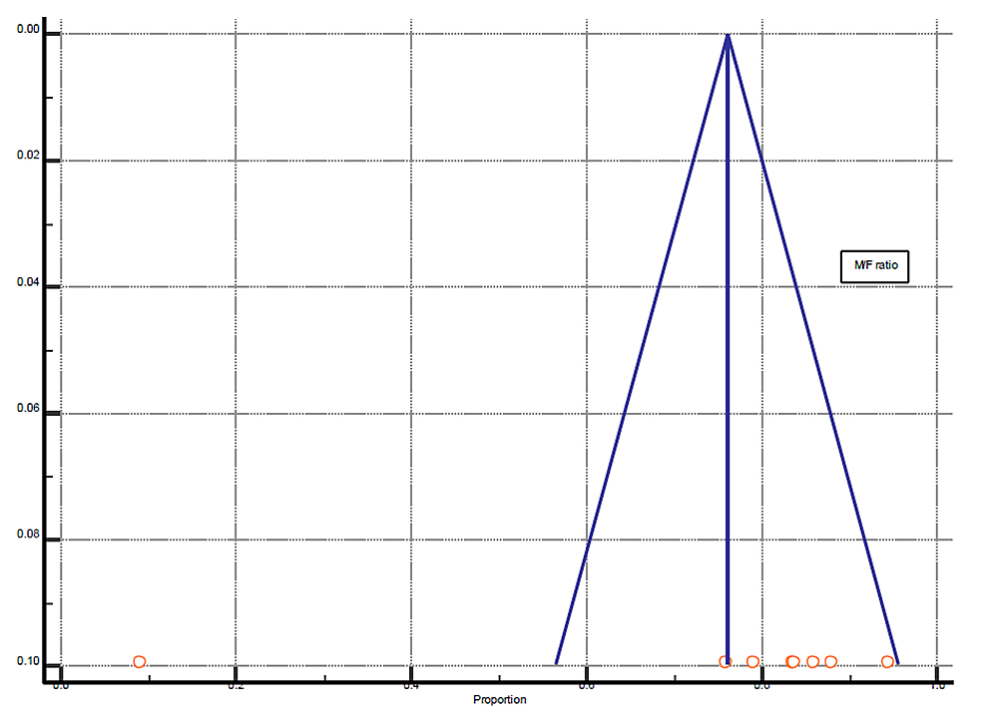
Funnel plot presentation of male/female (M/F) ratio

Among all selected studies, few studies were based on male-only or female-only CH patients. However, we included only those studies for statistical analysis which reported male-to-female prevalence among CH patients. Eight studies fulfilled the criteria reported with P = 0.0005 and 72.96% I^2^ (inconsistency) (Figures 7-8, Table 5).

**Table 5:** Test for Heterogeneity of Male/Female Ratio CI: confidence interval; DF: degrees of freedom; I^2^: I-squared value

Q-value	DF	Significance level	I^2^ (inconsistencies)	95% CI for I^2^	Total (fixed effects)	Total (random effects)
25.8867	7	P < 0.0005	72.96%	44.78 to 86.76	80.437 to 85.712	77.963 to 87.849

**Figure 9:**
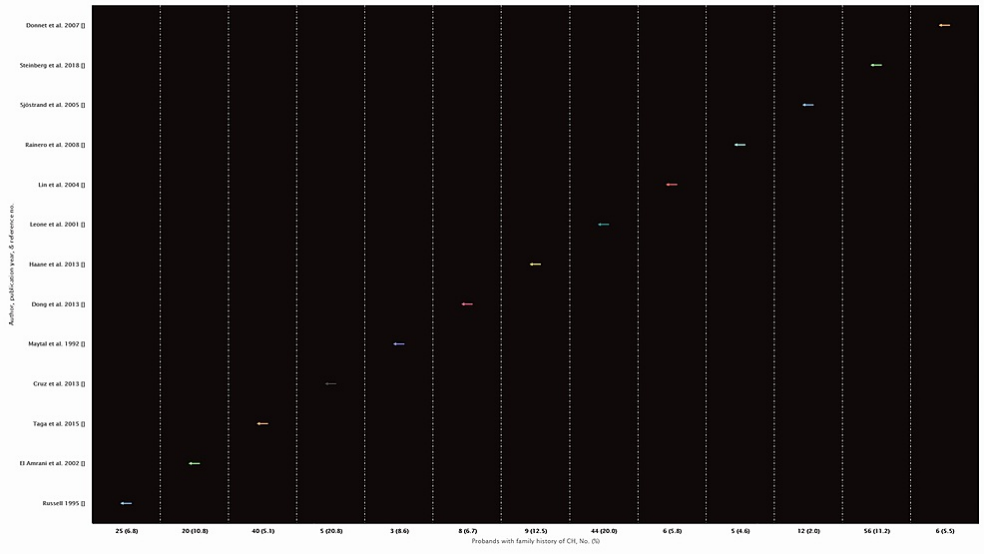
Presentation of probands with family history [11-22, 34]

The presentation of probands with family history is shown in Figure 9.

Discussion

The previous two decades contributed a great deal to the extensive scientific data on lifestyle habits, associated risk factors, and genetic linkage in association with CH and its severity. In this systemic review and meta-analysis, we list the most crucial associated factors of CH, including smoking, drinking/alcohol consumption, male-to-female ratio, head trauma, and genetic evidence of family history. A very recent review on lifestyle modification reported evidence of tobacco consumption and alcohol drinking as promoting factors of CH [40]. Schürks et al. reported evidence a decade ago that lifestyle habits, including smoking and alcohol drinking, were significantly linked with CH [4].

Out of 32 studies, 14 studies from the 1990s and 2021 reported the link between smoking and CH. These studies were based on retrospective data, surveys, and cross-sectional studies comprising 3,538 CH patients from all around the world. Among all, Rozen et al. reported a large-scale study with 1,134 CH patients with an 88% tobacco prevalence. This significantly linked tobacco consumption and CH with a 2.7:1 male-to-female ratio [33]. Tobacco consumption has been reported as one of the leading risk factors; it is not only linked with CH but this habit is also associated with the transition of disease severity from episodic CH to chronic CH [33]. Studies by Steinberg et al., Al-Hashel et al., and Ferrari et al. evidently support disease severity with tobacco consumption [22, 25, 28]. Chung et al. reported another aspect that clinical presentation of the disease did not differ between smokers and nonsmokers [24]. However, the history of tobacco consumption is strongly associated with the age of the disease onset. Some studies were based on only male or female-only participants and also concluded that smoking was a statistically significant indicator, was critically linked with CH in both genders, and strongly recommended healthy lifestyle habits among CH patients [30-32]. Alcohol consumption/alcohol drinking was another lifestyle habit that was evaluated and reported in CH patients. Five studies comprising 539 CH patients reported a drinking history and alcohol consumption. All studies were based on retrospective data analysis and reported drinking habits among CH diagnosed patients. However, these studies did not report quantification of alcohol, such as the number of wine glasses daily or weekly. Schürks et al. supported the linkage of alcohol consumption and CH, based on studies reported a decade ago [4]. There have been many headache forms identified which have been initiated by alcohol intake. The International Classification of Headache Disorders (ICHD-3 beta) extensively classified headaches and their trigger factors and reported that alcohol intake contributes to the activation of CH [41]. Studies of alcohol consumption were reported from India, Korea, Kuwait, Taiwan, and Japan. Alcohol consumption frequency varied among these studies; Western countries possibly have different numbers, and cultural diversity might be the reason. Sjöstrand et al. also supported the connection of poor lifestyle habits with CH [37]. However, a study by Schürks et al. from Germany reported less alcohol intake in CH patients than the general population and almost the same predictors of alcohol consumption among these two groups [38]. This study was based on 246 patients.

Eight studies among the selected studies reported the prevalence of gender discrimination in CH patients [2, 8, 19, 24-28]. The selected studies from 2004 to 2021 demonstrate the predominance of males among CH patients. A study by Manzoni in 1998 reported a descriptive analysis of gene ratio alteration before the 1960s till the 1990s because of bad lifestyle habits, such as smoking [39]. A recent study by Allena et al. reported the difference in symptom occurrence between males and females. Females had more painful episodes with extended mean attack duration [7]. This difference is possibly connected to sex hormones. However, smoking habits were largely seen in the male patient group. Head trauma and CH association was the least reported factor. Grangeon et al. recently reported an extensive study on post-traumatic headache (PTH) in 553 CH patients [8]. This study revealed 26 patients with post-traumatic headache and linked this to the chronic and intractable CH form. In 2010, Lambru et al. reported a study based on 200 patients where traumatic injuries were more often seen in CH patients with smoking and alcohol drinking [36]. These lifestyle habits are not only linked with CH and severity but might also increase the possibility of traumatic injuries. 

Genetic linkage and family history of CH is the most frequent and explicit factor reported. Waung et al. reported an extensive systemic review and reported it as a genetic pathology having layers of genetic patterns [3]. This study reported the high female prevalence among familial cases, which needs to be explored. 

Coffee consumption and CH connection might be another risk factor. A study by Lambru et al. linked this risk factor with frequent head injuries in CH patients, along with smoking and alcohol drinking [36]. Manzoni also highlighted the connection between coffee consumption and CH in his findings in 1998 [39].

This systemic review and meta-analysis were limited in many aspects. This study does not report all CH-associated risk factors, such as coffee consumption and age. We did not report the pathophysiology of reported risk factors, including smoking, alcohol drinking, sex discrimination, head injury, and familial cases.

## Conclusions

This systemic review meta-analysis revealed the overall picture of all important associated risk factors of cluster headache. Smoking and alcohol drinking were strongly linked to cluster headaches and their severity. This also leads to an increase in the possibility of head trauma. Male prevalence was reportedly high in our selected studies; however, disease presentation might differ because of the difference in sex hormones. Cluster head and genetic linkage were found connected; a higher female ratio was seen in familial cases.

## References

[1] O'Connor E, Simpson BS, Houlden H, Vandrovcova J, Matharu M (2020). Prevalence of familial cluster headache: a systematic review and meta-analysis. J Headache Pain.

[2] Moon HS, Park JW, Lee KS (2017). Clinical features of cluster headache patients in Korea. J Korean Med Sci.

[3] Waung MW, Taylor A, Qualmann KJ, Burish MJ (2020). Family history of cluster headache: a systematic review. JAMA Neurol.

[4] Schürks M, Diener HC (2008). Cluster headache and lifestyle habits. Curr Pain Headache Rep.

[5] Joshi S, Rizzoli P, Loder E (2017). The comorbidity burden of patients with cluster headache: a population-based study. J Headache Pain.

[6] Dueland AN (2015). Headache and alcohol. Headache.

[7] Allena M, De Icco R, Sances G (2019). Gender differences in the clinical presentation of cluster headache: a role for sexual hormones?. Front Neurol.

[8] Grangeon L, O'Connor E, Chan CK, Akijian L, Pham Ngoc TM, Matharu MS (2020). New insights in post-traumatic headache with cluster headache phenotype: a cohort study. J Neurol Neurosurg Psychiatry.

[9] Higgins JP, Altman DG, Gøtzsche PC (2011). The Cochrane Collaboration's tool for assessing risk of bias in randomised trials. BMJ.

[10] Swartz MK (2011). The PRISMA statement: a guideline for systematic reviews and meta-analyses. J Pediatr Health Care.

[11] Russell MB, Andersson PG, Thomsen LL (1995). Familial occurrence of cluster headache. J Neurol Neurosurg Psychiatry.

[12] El Amrani M, Ducros A, Boulan P (2002). Familial cluster headache: a series of 186 index patients. Headache.

[13] Taga A, Russo M, Manzoni GC, Torelli P (2015). Familial cluster headache in an Italian case series. Neurol Sci.

[14] Cruz S, Lemos C, Monteiro JM (2013). Familial aggregation of cluster headache. Arq Neuropsiquiatr.

[15] Maytal J, Lipton RB, Solomon S, Shinnar S (1992). Childhood onset cluster headaches. Headache.

[16] Dong Z, Di H, Dai W (2013). Clinical profile of cluster headaches in China - a clinic-based study. J Headache Pain.

[17] Haane DY, Plaum A, Koehler PJ, Houben MP (2016). High-flow oxygen therapy in cluster headache patients has no significant effect on nociception specific blink reflex parameters: a pilot study. J Headache Pain.

[18] Leone M, Russell MB, Rigamonti A (2001). Increased familial risk of cluster headache. Neurology.

[19] Lin KH, Wang PJ, Fuh JL, Lu SR, Chung CT, Tsou HK, Wang SJ (2004). Cluster headache in the Taiwanese -- a clinic-based study. Cephalalgia.

[20] Rainero I, Gallone S, Rubino E (2008). Haplotype analysis confirms the association between the HCRTR2 gene and cluster headache. Headache.

[21] Sjöstrand C, Russell MB, Ekbom K, Hillert J, Waldenlind E (2005). Familial cluster headache. Is atypical cluster headache in family members part of the clinical spectrum?. Cephalalgia.

[22] Steinberg A, Fourier C, Ran C, Waldenlind E, Sjöstrand C, Belin AC (2018). Cluster headache - clinical pattern and a new severity scale in a Swedish cohort. Cephalalgia.

[23] Bhargava A, Pujar GS, Banakar BF, Shubhakaran K, Kasundra G, Bhushan B (2014). Study of cluster headache: a hospital-based study. J Neurosci Rural Pract.

[24] Chung PW, Kim BS, Park JW (2021). Smoking history and clinical features of cluster headache: results from the Korean Cluster Headache Registry. J Clin Neurol.

[25] Al-Hashel J, Ibrahim I, Youssry D, Ahmed SF, Goadsby P (2019). Cluster headache in Kuwait: a hospital-based study. Front Neurol.

[26] Ko CA, Lin GY, Ting CH (2021). Clinical features of cluster headache: a hospital-based study in Taiwan. Front Neurol.

[27] Imai N, Kitamura E (2019). Differences in clinical features of cluster headache between drinkers and nondrinkers in Japan. PLoS One.

[28] Ferrari A, Zappaterra M, Righi F (2013). Impact of continuing or quitting smoking on episodic cluster headache: a pilot survey. J Headache Pain.

[29] Lund N, Petersen A, Snoer A, Jensen RH, Barloese M (2019). Cluster headache is associated with unhealthy lifestyle and lifestyle-related comorbid diseases: results from the Danish Cluster Headache Survey. Cephalalgia.

[30] Hannerz J (1997). Symptoms and diseases and smoking habits in female episodic cluster headache and migraine patients. Cephalalgia.

[31] Manzoni GC (1999). Cluster headache and lifestyle: remarks on a population of 374 male patients. Cephalalgia.

[32] Levi R, Edman GV, Ekbom K, Waldenlind E (1992). Episodic cluster headache II: high tobacco and alcohol consumption in males. Headache.

[33] Rozen TD (2018). Cluster headache clinical phenotypes: Tobacco nonexposed (never smoker and no parental secondary smoke exposure as a child) versus tobacco‐exposed: results from the United States Cluster Headache Survey. Headache.

[34] Donnet A, Lanteri-Minet M, Guegan-Massardier E (2007). Chronic cluster headache: a French clinical descriptive study. J Neurol Neurosurg Psychiatry.

[35] Torelli P, Manzoni GC (2003). Clinical observations on familial cluster headache. Neurol Sci.

[36] Lambru G, Castellini P, Manzoni GC, Torelli P (2010). Mode of occurrence of traumatic head injuries in male patients with cluster headache or migraine: is there a connection with lifestyle?. Cephalalgia.

[37] Sjöstrand C, Russell MB, Ekbom K, Waldenlind E (2010). Familial cluster headache: demographic patterns in affected and nonaffected. Headache.

[38] Schürks M, Kurth T, Knorn P, Pageler L, Diener HC (2006). Predictors of hazardous alcohol consumption among patients with cluster headache. Cephalalgia.

[39] Manzoni GC (1998). Gender ratio of cluster headache over the years: a possible role of changes in lifestyle. Cephalalgia.

[40] Raucci U, Boni A, Evangelisti M (2020). Lifestyle modifications to help prevent headache at a developmental age. Front Neurol.

[41] (2018). Headache Classification Committee of the International Headache Society (IHS) The International Classification of Headache Disorders, 3rd edition. Cephalalgia.

